# Peritoneal Metastasis Mimicking Chemotherapy-Induced Complications in Lung Adenocarcinoma: A Diagnostic Challenge of a Case Report

**DOI:** 10.7759/cureus.82530

**Published:** 2025-04-18

**Authors:** Ryuichi Ohta, Kaoru Tanaka, Masayuki Miyata, Junko Tanizaki, Hidetoshi Hayashi

**Affiliations:** 1 Community Care, Unnan City Hospital, Unnan, JPN; 2 Medical Oncology, Kindai University Faculty of Medicine, Sayama, JPN; 3 Oncology, Kindai University Faculty of Medicine, Sayama, JPN

**Keywords:** diagnosis, differential, docetaxel, drug-related side effects and adverse event, neoplasm metastasis, non-small cell lung cancer, peritoneal neoplasms

## Abstract

We report a case of a 64-year-old man with advanced non-small cell lung cancer (NSCLC) who developed peritoneal metastasis during systemic treatment. Initially diagnosed with lung adenocarcinoma with pleural dissemination and bone metastases, he received carboplatin, pemetrexed, and pembrolizumab, followed by docetaxel due to clinical progression. While primary lung lesions responded to docetaxel, the patient developed new-onset abdominal pain and ascites. Radiologic findings suggested peritoneal thickening, raising suspicion for either docetaxel-induced toxicity or disease progression. Given the rarity of peritoneal metastasis in NSCLC and concurrent treatment response elsewhere, drug-induced complications were initially considered. However, worsening symptoms and further imaging prompted cytological evaluation of ascitic fluid, which confirmed metastatic adenocarcinoma consistent with lung origin. This case highlights the diagnostic challenge of distinguishing treatment-related adverse events from disease progression, especially in patients presenting with nonspecific abdominal symptoms during therapy. Clinicians should maintain a high index of suspicion for uncommon metastatic sites when new symptoms arise, even in the setting of apparent response at the primary site.

## Introduction

Non-small cell lung cancer (NSCLC) is a heterogeneous group of lung malignancies that often progress rapidly and metastasize to various organs, contributing to its high morbidity and mortality [1]. Accurate identification of metastatic sites is critical in managing advanced NSCLC, as it directly influences treatment decisions, prognostic assessment, and appropriate palliative care [2].

Although rare, peritoneal metastasis from NSCLC poses a diagnostic challenge due to its nonspecific presentation and overlap with treatment-related complications [3]. While uncommon in NSCLC, it must be carefully distinguished from treatment-related complications, such as ascites, and peritoneal changes induced by chemotherapeutic agents [4]. Docetaxel, a commonly used second-line agent in NSCLC, has been associated with such complications, including fluid retention and peritoneal inflammation [5].

Herein, we report a case of a 64-year-old patient with advanced NSCLC presenting with bone metastases and pleural effusion, who subsequently developed rapid-onset ascites and peritoneal changes. Despite stable disease at the primary site, the emergence of abdominal symptoms initially raised suspicion for docetaxel-induced toxicity [6]. However, further clinical evaluation revealed peritoneal metastasis. This case highlights the diagnostic complexity in distinguishing between drug-related adverse events and disease progression in NSCLC, and we discuss practical approaches to this differential diagnosis based on clinical experience and existing literature.

## Case presentation

A 64-year-old man with a history of hypertension and a 40-pack-year smoking history presented to our respiratory department with a two-month history of persistent cough. He had initially visited a local clinic approximately 80 days before presentation, where a chest X-ray showed an infiltrative shadow in the right lung field. Chest computed tomography (CT) revealed a mass in the right middle and lower lobes, prompting referral to our hospital for further evaluation. His past medical history was hypertension and dyslipidemia. His medication was amlodipine 5 mg daily. He did not have any family history of malignancy, travel history to other countries, or exposure to patients with tuberculosis within 10 years.

Upon arrival, his vital signs were as follows: temperature 36.4 °C, blood pressure 139/68 mmHg, heart rate 87 beats per minute (regular), respiratory rate 16 breaths per minute, and oxygen saturation 97% on room air. His height was 164 cm, weight 67 kg, and BMI 24.9. He was alert and fully oriented with an Eastern Cooperative Oncology Group Performance Status (ECOG PS) of 0. Physical examination revealed bilateral crackles on lung auscultation. There was no cervical lymphadenopathy, anemia, jaundice, edema, abdominal tenderness, or neurological deficit.

Initial laboratory tests revealed elevated carcinoembryonic antigen (CEA) and cytokeratin 19 fragment (CYFRA) levels (Table 1).

**Table 1:** Initial laboratory data of the patient CEA, carcinoembryonic antigen; CRP, C-reactive protein; CYFRA, cytokeratin 19 fragment; Ig, immunoglobulin; ProGRP, pro-gastrin-releasing peptide

Parameter	Level	Reference
White blood cells	6.56	3.5–9.1 × 10^3^/μL
Neutrophils	86.7	44.0–72.0%
Lymphocytes	5.7	18.0–59.0%
Hemoglobin	15.1	11.3–15.2 g/dL
Mean corpuscular volume	89.1	79.0–100.0 fl
Platelets	27.2	13.0–36.9 × 10^4^/μL
Total protein	6.6	6.5–8.3 g/dL
Albumin	4.2	3.8–5.3 g/dL
Aspartate aminotransferase	20	8–38 IU/L
Alanine aminotransferase	27	4–43 IU/L
Lactate dehydrogenase	186	121–245 U/L
Blood urea nitrogen	17	8–20 mg/dL
Creatinine	0.70	0.40–1.10 mg/dL
Serum Na	142	135–150 mEq/L
Serum K	4.2	3.5–5.3 mEq/L
Serum Cl	107	98–110 mEq/L
CRP	0.135	<0.30 mg/dL
CEA	400	0-5.0 ng/mL
CYFRA	5.4	0–3.5 ng/mL
ProGRP	47	<50 pg/mL
Urine test	-	-
Leukocyte	Negative	Negative
Protein	Negative	Negative
Blood	Negative	Negative

**Figure 1:**
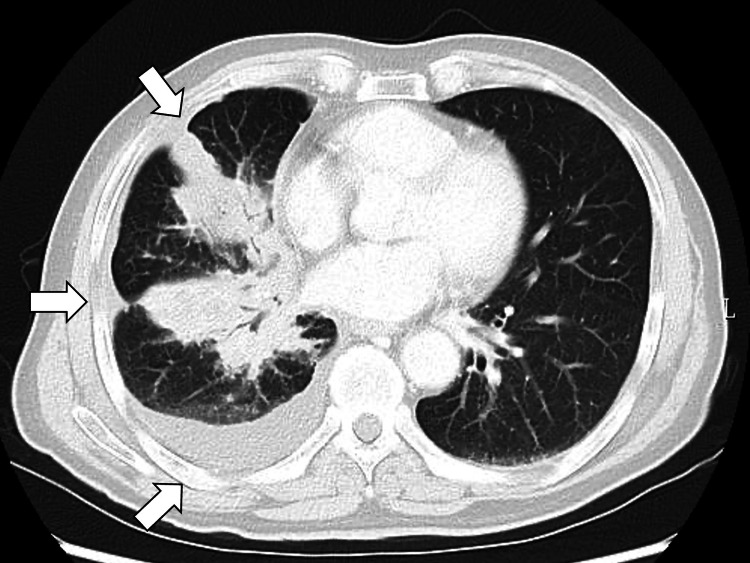
Chest-to-pelvis computed tomography showing a right lower lobe to the hilar mass with pleural thickening and right pleural effusion, suggesting carcinomatous lymphangitis and pleural dissemination (white arrows)

Chest-to-pelvis CT showed a right lower lobe to the hilar mass with pleural thickening and right pleural effusion, suggesting carcinomatous lymphangitis and pleural dissemination (Figure 1).

**Figure 2:**
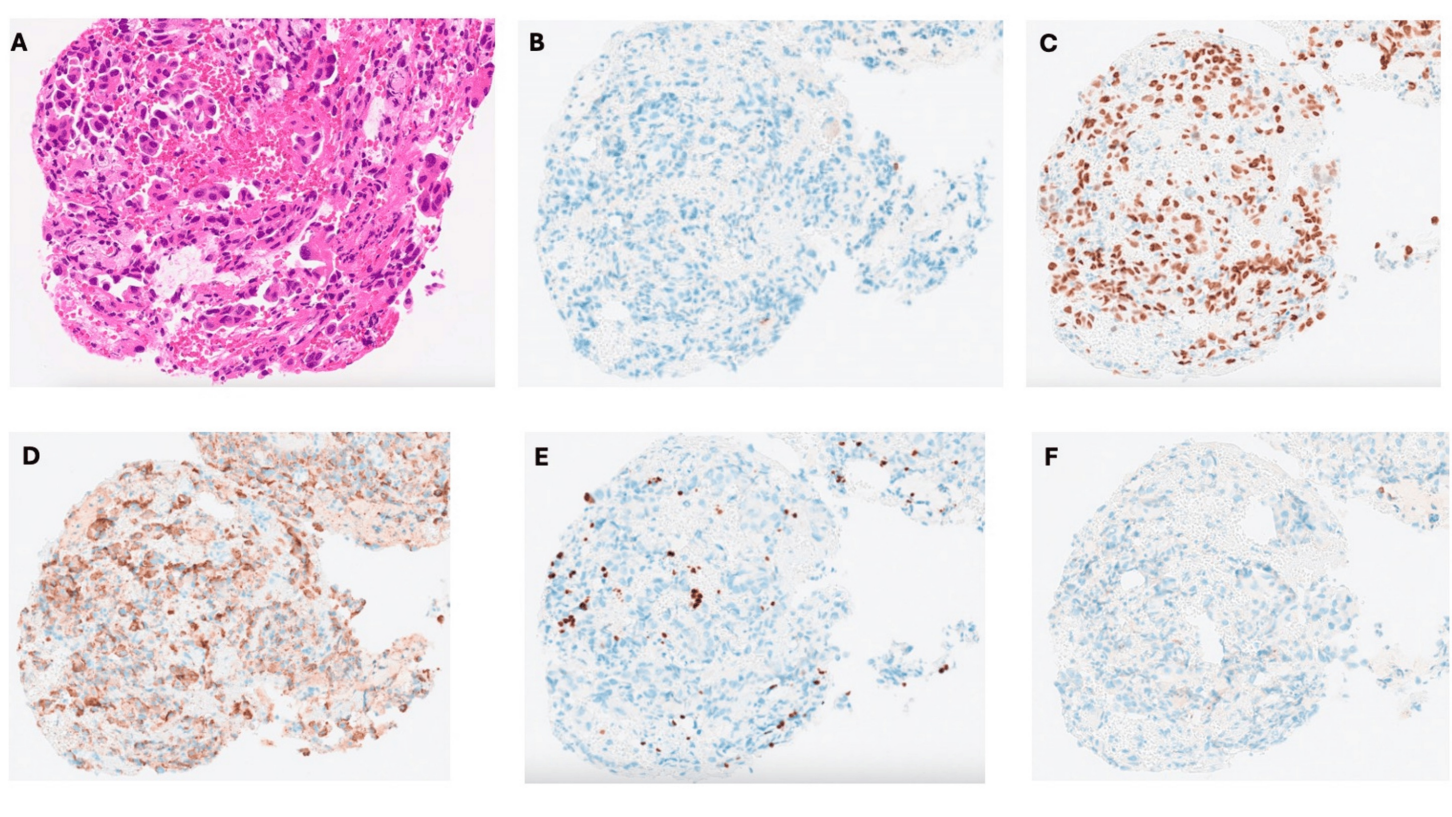
The immunopathological investigation of the tissues from the bronchoscopy showed large, atypical cells scattered or in small clusters in hematoxylin & eosin stain (A) Immunohistochemistry was positive for thyroid transcription factor-1 (B) and Napsin A (D) and negative for chromogranin A (B), p40 (E), and synaptophysin (F), consistent with adenocarcinoma.

Bronchoscopic biopsy revealed numerous atypical cells with increased chromatin, high nuclear/cytoplasmic (N/C) ratio, prominent nucleoli, and cellular pleomorphism. The immunopathological investigation showed that large, atypical cells were scattered or in small clusters. Immunohistochemistry was positive for Napsin A and thyroid transcription factor-1 (TTF-1) and negative for cytokeratin (CK) 5/6, p40, synaptophysin, and chromogranin A, consistent with adenocarcinoma (Figure 2).

Genetic analysis showed no actionable driver mutations. Programmed death-ligand (PD-L) 1 immunohistochemistry was negative with a tumor proportion score (TPS) of <1%.

The patient was diagnosed with metastatic lung adenocarcinoma (driver mutation-negative, PD-L1 negative). On Day 37, treatment was initiated with carboplatin (area under the curve (AUC) 5), pemetrexed (100 mg/m²), and pembrolizumab (200 mg/body) in three-week cycles. After two cycles, chest CT revealed stable disease but new findings suggestive of right pleural effusion and carcinomatous lymphangitis. Due to worsening symptoms, clinical progression (clinical PD) was suspected, and treatment was switched to docetaxel (75 mg/m^2^) starting on Day 98. Prophylactic pegfilgrastim was administered on Day 99.

**Figure 3:**
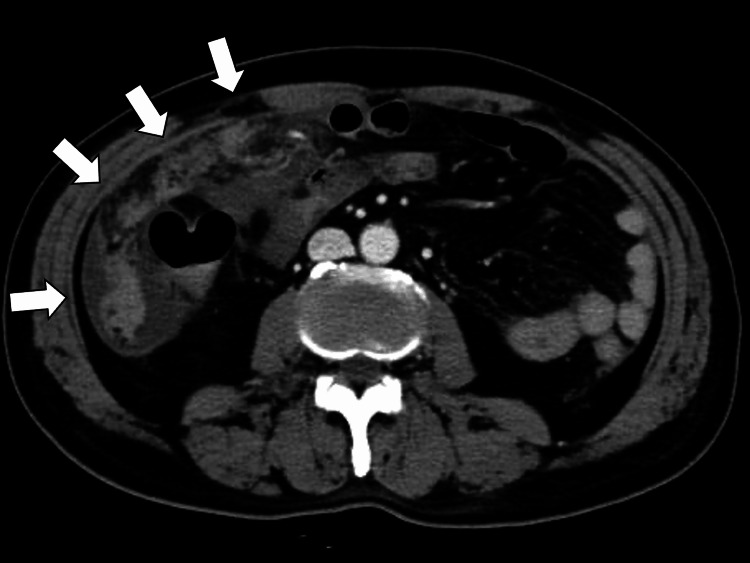
Abdominal computed tomography revealing newly developed ascites and peritoneal thickening (white arrows)

A follow-up CT on Day 124 after two cycles of docetaxel showed tumor shrinkage, and the patient's symptoms had improved. However, during a follow-up visit on Day 180, the patient complained of worsening abdominal pain. The abdominal CT revealed newly developed ascites and peritoneal thickening (Figure 3).

**Figure 4:**
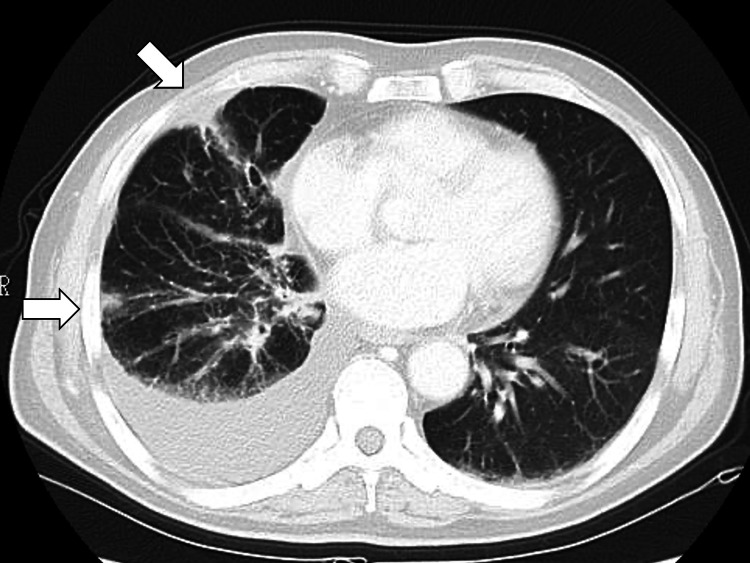
Chest computed tomography showing continued response at the primary site of the lung (white arrows)

The chest CT also showed continued response at the primary site without the significant elevation of tumor markers (Figure 4).

In the differentiation between docetaxel-induced side effects and peritoneal metastasis of NSCLC, considering the prevalence and clinical course of the NSCLC of the patient, docetaxel-induced side effects were suspected. Thus, the same chemotherapy was continued under close observation.

**Figure 5:**
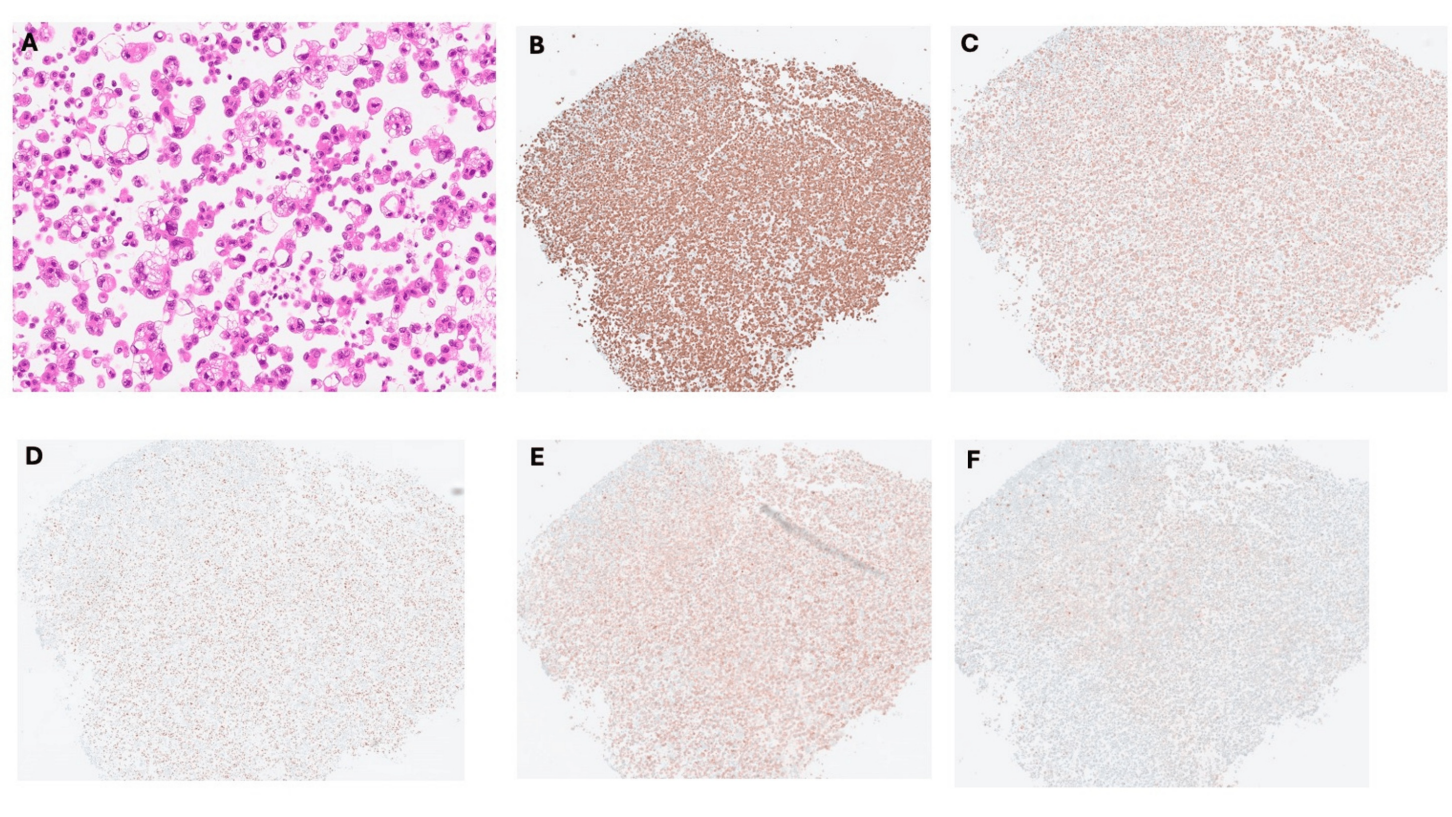
Cytopathological examination of ascitic fluid showed cell clusters with nuclear enlargement, some forming glandular structures on hematoxylin and eosin staining (A) The immunopathological investigation of the ascites tissues showed that immunohistochemistry was positive for anti-epithelial (AE)1/AE3 (B), thyroid transcription factor-1 (C), Napsin A (D), Claudin-4 (E), and carcinoembryonic antigen (CEA) (F), consistent with adenocarcinoma.

Due to persistent symptoms, as peritoneal metastasis from NSCLC was suspected, a repeated CT showed the exacerbation of peritoneal thickening and mass formation. Cytopathological examination of ascitic fluid showed cell clusters with nuclear enlargement, some forming glandular structures. Immunohistochemistry was positive for anti-epithelial (AE)1/AE3, CEA, Claudin-4, Napsin A, and TTF-1, with weak Wilms tumor (WT)-1 positivity, consistent with adenocarcinoma (Figure 5).

Thus, on Day 192, disease progression was confirmed. The chemotherapy was discontinued, and the patient was hospitalized for symptom management. Pain control was achieved with morphine, and, per the patient’s wishes, he was transferred to a palliative care facility for end-of-life care.

## Discussion

This case showed the rapid progression of NSCLC even during the administration of chemotherapy, highlighting the aggressive nature of the disease in specific clinical contexts. Although systemic therapies, such as platinum-based regimens or immune checkpoint inhibitors, have improved survival in patients with advanced NSCLC, resistance develops in many cases, leading to treatment failure and disease dissemination [4,5]. In this patient, the emergence of abdominal symptoms during chemotherapy raised initial suspicion for a gastrointestinal or treatment-related complication. However, the subsequent diagnosis of peritoneal metastasis underscores how subtle signs may herald disease progression despite ongoing therapy.

Peritoneal metastasis from NSCLC is rare, with a 1-2% reported frequency in autopsy studies and clinical cohorts [7,8]. Recent case series have drawn attention to this uncommon but significant pattern of disease spread, especially in patients with adenocarcinoma histology and those previously exposed to multiple lines of treatment [9]. In the current case, peritoneal involvement was confirmed only after a rapid clinical decline, emphasizing the importance of maintaining a broad differential diagnosis in the presence of new abdominal complaints in patients undergoing treatment for lung cancer.

A key learning point from this case is that resistance to chemotherapy can be a critical factor in facilitating distant metastases, including to unusual sites such as the peritoneum. Mechanistically, resistance may arise due to mutations in driver genes, epithelial-mesenchymal transition, or the tumor microenvironment's adaptive changes under therapeutic pressure [4,10,11]. In such scenarios, malignant cells may acquire enhanced migratory capabilities, resulting in metastases to less common anatomical compartments [12].

Furthermore, the differentiation between chemotherapy-related complications and cancer progression poses a diagnostic challenge in clinical practice. For instance, gastrointestinal toxicities such as ileus, colitis, or abdominal pain may arise from cytotoxic agents or immune-related adverse events [13,14]. In contrast, peritoneal carcinomatosis may present with similar nonspecific symptoms, including bloating, pain, or ascites [15]. As demonstrated in this case, a careful assessment of the clinical course, prevalence of potential etiologies, and radiological findings is essential to guide appropriate diagnosis and timely intervention. In addition, recent advances in oncology have enabled the detection of cancer progression through the analysis of tumor markers and circulating tumor DNA (ctDNA) via blood-based assays [16]. These cutting-edge microbiological techniques facilitate the monitoring of disease dynamics and allow for the non-invasive assessment of cancer progression in various clinical scenarios.

Ultimately, this case highlights the need for vigilance when interpreting new symptoms in patients with advanced NSCLC, particularly those with a history of treatment resistance or prolonged therapy [17,18]. Future studies are warranted to better characterize the clinical features and risk factors for peritoneal metastasis in NSCLC, which may assist in earlier recognition and optimized management of such cases.

## Conclusions

This case underscores the importance of maintaining a broad differential diagnosis when evaluating new abdominal symptoms in patients with advanced non-small cell lung cancer undergoing chemotherapy. Peritoneal metastasis, though rare, should be considered even when primary tumor sites show a response to treatment. The overlap between chemotherapy-induced adverse effects and metastatic disease can obscure timely diagnosis, potentially delaying appropriate management. Careful assessment of symptom progression, imaging findings, and pathological confirmation through ascitic fluid analysis is crucial for accurate diagnosis. Clinicians should remain vigilant for atypical metastatic patterns to ensure optimal patient care and timely transition to palliative support when needed. However, this report is limited by the absence of genetic or molecular analysis, which could have provided further insight into the mechanisms underlying atypical metastatic spread. Future studies should better explore the molecular characteristics of such cases to understand predictors of peritoneal dissemination in lung cancer.
